# Fine scale transitions of the microbiota and metabolome along the gastrointestinal tract of herbivorous fishes

**DOI:** 10.1186/s42523-022-00182-z

**Published:** 2022-05-23

**Authors:** Wesley J. Sparagon, Emily C. Gentry, Jeremiah J. Minich, Lisa Vollbrecht, Lieve M. L. Laurens, Eric E. Allen, Neil A. Sims, Pieter C. Dorrestein, Linda Wegley Kelly, Craig E. Nelson

**Affiliations:** 1 Daniel K. Inouye Center for Microbial Oceanography: Research and Education, Department of Oceanography and Sea Grant College Program, University of Hawaiʻi at Mānoa, 1950 East West Road, Honolulu, HI 96822 USA; 2 Skaggs School of Pharmacy and Pharmaceutical Sciences, University of California San Diego, La Jolla, CA USA; 3 Collaborative Mass Spectrometry Innovation Center, Skaggs School of Pharmacy and Pharmaceutical Sciences, University of California San Diego, La Jolla, CA USA; 4 The Plant Molecular and Cellular Biology Laboratory, The Salk Institute for Biological Studies, La Jolla, CA USA; 5 Ocean Era, Natural Energy Laboratory of Hawai’i, Kailua-Kona, HI USA; 6 Biosciences Center, Bioenergy Science and Technology Directorate, National Renewable Energy Laboratory, Golden, CO USA; 7 Molecular Biology Section, Division of Biological Sciences, University of California San Diego, La Jolla, CA USA; 8 Marine Biology Research Division, Scripps Institution of Oceanography, University of California San Diego, La Jolla, CA USA

**Keywords:** Fish gastrointestinal tract microbiota metabolome transitions

## Abstract

**Background:**

Gut microorganisms aid in the digestion of food by providing exogenous metabolic pathways to break down organic compounds. An integration of longitudinal microbial and chemical data is necessary to illuminate how gut microorganisms supplement the energetic and nutritional requirements of animals. Although mammalian gut systems are well-studied in this capacity, the role of microbes in the breakdown and utilization of recalcitrant marine macroalgae in herbivorous fish is relatively understudied and an emerging priority for bioproduct extraction. Here we use a comprehensive survey of the marine herbivorous fish gut microbial ecosystem via parallel 16S rRNA gene amplicon profiling (microbiota) and untargeted tandem mass spectrometry (metabolomes) to demonstrate consistent transitions among 8 gut subsections across five fish of the genus of *Kyphosus*.

**Results:**

Integration of microbial phylogenetic and chemical diversity data reveals that microbial communities and metabolomes covaried and differentiated continuously from stomach to hindgut, with the midgut containing multiple distinct and previously uncharacterized microenvironments and a distinct hindgut community dominated by obligate anaerobes. This differentiation was driven primarily by anaerobic gut endosymbionts of the classes *Bacteroidia* and *Clostridia* changing in concert with bile acids, small peptides, and phospholipids: bile acid deconjugation associated with early midgut microbiota, small peptide production associated with midgut microbiota, and phospholipid production associated with hindgut microbiota.

**Conclusions:**

The combination of microbial and untargeted metabolomic data at high spatial resolution provides a new view of the diverse fish gut microenvironment and serves as a foundation to understand functional partitioning of microbial activities that contribute to the digestion of complex macroalgae in herbivorous marine fish.

**Supplementary Information:**

The online version contains supplementary material available at 10.1186/s42523-022-00182-z.

## Background

Multicellular organisms exist in association with a myriad of symbiotic microorganisms including viruses, bacteria, archaea, protists, and fungi, collectively termed “microbiota” [1]. These host-microbiota associations are now recognized as taxonomically widespread and critical for maintaining host function [2–6]. Extensive research has highlighted the important roles microbes play in host biology, especially in the gastrointestinal tract, and it has been shown that gut microbiota, primarily made up of bacteria and archaea, are essential in the maintenance of normal host function [7]. Gastrointestinal tracts provide an ideal habitat for microbiota, which can grow to concentrations upwards of 10^11^ cells per mL [8]. Gut microbes reciprocally provide a variety of services to the host, namely aiding in the digestion of food by breaking down molecules which the host cannot. There is a diverse array of metabolic digestive processes mediated by microbiota including the anaerobic fermentation of organic compounds to yield short-chain fatty acids (SCFAs) [9–12]. These metabolic products are then accessible to the host and are used as sources of energy, nutrition and signaling molecules in host-microbe interactions [13].

Gut microbiota dynamics have been heavily studied in terrestrial organisms such as humans and ruminants, yet research is relatively lacking in aquatic vertebrates such as fishes. Recent research has begun to shed light on this topic but with a narrow focus on a few commercially relevant aquaculture fish such as salmonids and carp [14]. Due to their ecological and biotechnological relevance, there is a growing interest in herbivorous fish gut microbiota and their digestive capabilities. Marine herbivorous fish consume seagrass and/or marine algae, regulating the abundance of benthic algae and helping maintain the health of the entire ecosystem [15, 16]. It is thought that the gut microbiota of herbivorous fishes, hereafter referring specifically to bacterial and archaeal symbionts, plays a critical role in digestion of algal molecules for the fish host [17]. This process is not only of ecological importance but is also relevant to the development of marine algae as a novel source of energy and metabolites for humans; microbial processes that deconstruct algal compounds into useful metabolites in herbivorous fish guts can serve as a model for future ex situ bioreactor systems [18, 19].

Gut location is one of the strongest factors structuring marine herbivorous fish gut microbiota, with different gut sections hosting vastly different microbial communities [20–24]. Marine herbivorous fish guts are dominated by bacteria in the phyla *Proteobacteria*, *Bacteroidetes*, and *Firmicutes*, with dominant families including *Vibrionaceae* and *Clostridiaceae* [6, 21, 22]. These microbial communities are influenced not only by location within the gut but also by other factors including host phylogeny, fish age and life history traits, diet, and sample type (e.g. digesta vs. gut lumen) [6, 11, 21–24]. It has been suggested that microbial community differentiation along the gut facilitates distinct processes that aid in the stepwise digestion and utilization of algal biomass, yielding distinct chemistries in each of the gut sections [21]. Histological data from marine herbivorous fishes including members of Kyphosidae (*Kyphosus sydneyanus*) reveal direct evidence of morphological specialization along the gastrointestinal tract and altered absorptive modes indicative of the prominent roles of microbial metabolism of algae in the posterior gastrointestinal tract [25]. In parallel to morphological evidence, carbohydrase enzyme activity assays have revealed spatial variation in microbial contributions to the breakdown of starch, laminarian, carrageenan, alginate, and agarose, all of which were elevated in the posterior portions of the gut [26], as well as functional partitioning between endogenous breakdown of starch and exogenous (microbial) breakdown of structural carbohydrates [27].

The midgut and the hindgut of herbivorous fishes maintain distinct microbial communities, with the hindgut having a high abundance of anaerobic bacteria such as *Rikenellaceae*, *Ruminococcaceae*, *Clostridiaceae*, and *Desulfovibrionaceae* [28–31]. This has led researchers to hypothesize that marine herbivorous fish gut microbiota aid in the digestion of marine algae through anaerobic fermentative processes that resemble their terrestrial herbivorous counterparts. Large algal polysaccharides are first broken down to smaller constituents in the stomach and midgut, followed by anaerobic fermentation of these smaller constituents in the hindgut, yielding SCFAs that can then be utilized by the host fish [32]. Multiple studies have observed elevated levels of SCFAs in herbivorous fish hindguts, confirming the hindgut as the site of microbial anaerobic fermentation and production of SCFAs in this system [31, 33, 34].

Despite preliminary progress, much knowledge is lacking about the role fish gut microbiota play in the breakdown of algae beyond fermentation and SCFA production, including the extraction/production of other vital molecules such as lipids and amino acids [11, 35]. Unfortunately, most studies cannot elucidate the above processes due to limited spatial and metabolic sampling schemes. Spatial undersampling along the gastrointestinal tract neglects potential fine scale variation in gut dynamics that could shed light onto microbe-mediated deconstruction of algal biomass. Additionally, surveys of microbial members in the gut yields limited information about chemical transformations. Even targeted measurements of specific metabolites do not provide the unbiased, untargeted approach necessary to unravel the full scope of chemical changes occurring through the gut. Untargeted metabolomics provides an ideal avenue to profile chemical shifts through the gut and has been identified as a necessary component of future fish gut research [11]. When used in parallel with microbiota profiling, these two data streams can provide a more complete picture of changes within the gut, especially in regards to the breakdown of complex molecules, the chemical modification of small molecules, and which microbial communities and taxa might be responsible. Untargeted metabolomics via tandem mass spectrometry has already proven incredibly useful in mammalian gut studies, but to our knowledge has not previously been applied in fish gut studies [36].

In order to address these knowledge gaps, we conducted a 16S rRNA gene (microbiota) and untargeted LC–MS/MS (metabolomes) fine spatial-scale survey along the gastrointestinal tract of 5 marine herbivorous fishes in the genus *Kyphosus* (family Kyphosidae). Fishes in this genus, also known as Sea Chubs or Rudderfish, are tropical and sub-tropical fishes that offer an ideal system to study how gut microbes might aid in the digestion of marine algae in herbivorous fishes [37]. They are generally thought to be obligate herbivores and consume large amounts of macroscopic algae to supply their daily energy demands [37–39]. Subtropical/tropical waters contain seven Kyphosid species: *Kyphosus bigibbus, K. cinerascens, K. elegans, K. hawaiiensis, K. ocyurus, K. sectatrix and K. vaigiensis*. In Hawaiʻi, these species are called *nenue*; they are important food sources for subsistence fisheries and play critical roles in maintaining reef health by consuming macroalgae [40]. All nenue in Hawaiʻi appear to occupy similar rocky coastal habitats and maintain obligate herbivorous diets, with preferences for a variety of marine algae including turf, sargassum, and brown algae [37, 41]. The one noted exception appears to be *K. ocyurus*, which has a documented omnivorous diet including algae and zooplankton [41]. Studies have begun to shed light on how this genus of fish digests complex algal polysaccharides and how microbes might be involved. Most Kyphosids contain a morphologically distinct hindgut region in which elevated levels of SCFAs can be found [34, 39, 42]. Elevated microbial counts and putative anaerobic fermentative bacteria have also been observed in the hindgut region [34, 43]. This indicates that gut microbes likely play crucial roles in converting dietary macroalgae to usable energy for the fish host, with the site of microbial fermentation located in the hindgut. However, beyond the narrow focus on hindgut fermentation little is known about how gut microbes in nenue aid in the digestion of marine macroalgae.

In this study, nenue were used as a model to evaluate fine spatial-scale microbiota and metabolome changes in the herbivorous fish gut and to explore the implications of these changes for the microbially-mediated metabolism of macroalgae. Rather than evaluate microbiota/metabolome differences between fish species, life stages, etc., our aim was to sample a representative set of fishes to evaluate the consistency of longitudinal variation across the gut microbiome in the genus *Kyphosus*. Nenue digesta from five fish of the genus *Kyphosus* were sampled at 8 points along the gastrointestinal tract, from the stomach to the hindgut. High-throughput amplicon sequencing of the 16S rRNA gene and untargeted liquid chromatography tandem mass spectrometry (LC–MS/MS) were used to examine how microbial communities and metabolomes varied along the gut axis of nenue. These findings reveal spatial differentiation of herbivorous fish microbiota and metabolomes along the gastrointestinal tract and provide new insights into microbial taxa that contribute to the digestion and assimilation of marine macroalgae.

## Methods

### Sample collection and dissection

Fish of the genus *Kyphosus* were collected by local fishers in situ using a spear gun directly offshore of the Ocean Era facility at Keahole Point, Kona, Hawai’i Island, USA on June 11th and 12th, 2019 (19.7286, − 156.0619). Sea surface temperatures were 26.1 °C according to the NELHA surface seawater pipeline dataset [44] and fish were caught between roughly 1 to 10 m depth. Fish were transported within minutes to a shore-based sampling station and euthanized by pithing. Biometrics such as mass and fork length (the distance between the snout and the fork of the tail fin) were measured for each fish (Additional file 1). Fish were photographed to aid in identification [37] (Additional file 2). Record was taken if any trauma was sustained to the gastrointestinal tract while spearfishing. In cases where small portions of the gut were damaged, care was taken to avoid directly sampling those regions.

Fish guts were immediately transferred to a portable anaerobic chamber for dissection. Under anaerobic conditions, the belly of the fish was cut from the anus all the way up to the breast plate (sternum area) using a pair of field scissors. A scalpel was used to cut out the full gastrointestinal tract of the fish, which was removed from the fish by hand and tied off at the rostral and caudal section of the hindgut (defined as the terminal 4 cm of the GI tract) using dental floss. All dissections were done anaerobically and took 1–2 h each. After dissection the guts were moved out of the anaerobic chamber briefly for logistical reasons during subsampling and freezing of digesta for nucleic acid and metabolite analyses.

**Fig. 1:**
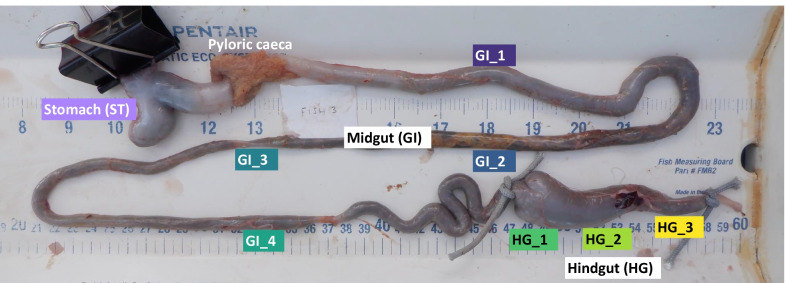
Fine-scale longitudinal sampling of the nenue gastrointestinal tract. The gut was subdivided and sampled in 8 gut subsections. Each gut subsection is colored according to its position in the gut with darker colors indicating more anterior and lighter colors indicating more posterior. Samples were taken from 5 fish of the *Kyphosus* genus

The gastrointestinal tracts were separated into three sections: Stomach (ST), Midgut (GI), and Hindgut (HG) (Fig. 1). These 3 sections were visually identified with the ST having a distinct morphology at the anterior end of the gastrointestinal tract and the HG exhibiting a distinct morphology at the posterior end. The GI (midgut) was identified as the long, uniform section of gastrointestinal tract between the clearly distinguishable pyloric caeca and HG. The GI was further divided into 4 equidistant subsections (GI 1–4) and the HG was divided into 3 equidistant subsections (HG 1–3), yielding a total of 8 subsection samples across the entire gastrointestinal tract (Fig. 1).

The digesta from each gut subsection was subsampled twice: once for DNA and once for metabolomes; both were immediately frozen − 20 °C. To accurately assess metabolomic and microbial shifts associated with algal digestion, we focused sampling efforts on digesta rather than gut wall (lumen). Each sampling point was cut open using a sterile razor blade, the digesta was homogenized with a sterile (autoclaved) wooden popsicle stick, and approximately 0.5 mL of digesta was collected into a 2 mL Qiagen DNeasy PowerSoil Kit Bead Tube containing agate crystals and Buffer A for downstream DNA analysis. These samples were inverted five times to mix and stored at − 20 °C until transport to the laboratory. Duplicate samples from each gut section/subsection were taken for metabolomics by scooping 0.5 mL of fish digesta into 2 mL sterile o-ring “cryovials” using sterile wooden popsicle sticks and stored at − 20 °C until transport to the laboratory. Samples were moved to − 80 °C within 2 days of collection until processing.

### DNA Extraction, Library Prep, 16S rRNA Gene Amplicon Sequencing, and Bioinformatics

Qiagen DNeasy PowerSoil Kit Bead Tubes containing the samples were thawed and extracted within 1 month of collection using the Qiagen DNeasy PowerSoil Kit (QIAGEN, Carlsbad, CA, United States) according to the manufacturers’ protocol. Amplicon sequencing of the V4 16S rRNA gene region was conducted on an Illumina MiSeq at the University of Hawaiʻi at Mānoa Advanced Studies in Genomics, Proteomics and Bioinformatics facility. Library preparation followed Kozich et al. 2013 [45]. In brief, a dual-index sequencing strategy was used to target the V4 region of the 16S rRNA gene region. 515F and 806R Earth Microbiome Project primers were used [46–49]. Amplicons were generated from a single round of PCR using dual index primers that include index sequences, Illumina spacers, Illumina adapters, and 16S rRNA gene template region (See Additional file 3 for PCR conditions). DNA extraction blanks and no-template control blanks were included as negative controls and mock communities (Zymobiomics, Zymo D6305 or D6306) were included as positive controls to enable discernment of contaminants from kits or processing [50–52]. Method blanks had substantially lower sequence read depth (median = 8957 reads/sample) than gut samples (median = 158,131 reads/sample), with samples ranging from 31,979 reads/sample to 347,727 reads/sample (Additional file 4). Total amplicons per sample were normalized to 25 ng using Charm Biotech Just-a-Plate 96 PCR Purification and Normalization (Charm Biotechnology, Cape Girardeau, MO, USA). Amplicons were pooled and sequenced using an Illumina MiSeq V3 600 paired-end cycle run at the University of Hawaiʻi at Mānoa Advanced Studies in Genomics, Proteomics and Bioinformatics facility. All samples were amplified and sequenced in triplicate technical replicates.

Raw paired fastq reads were processed to generate amplicon sequence variants (ASVs) [53, 54]. In brief, raw sequences were filtered, trimmed, and merged using the dada2 R package, triplicate technical replicates were merged bioinformatically after confirmation of replicability, and OTUs were defined as unique “amplicon sequence variants” by dada2 [55]. We used mothur [56] to align and annotate the sequences using the SILVA (release 132) SSU rRNA multiple sequence alignment database [57]. We removed all mitochondrial or chloroplast OTUs as well as sequences with no annotation at the domain level. Samples were subsampled at a depth of 50,000 sequences; 5 out of 45 samples were discarded due to insufficient read coverage. Lastly, we used the lulu R package to merge spurious ASVs and discarded ASVs with a total abundance of 2 reads or less across all samples [58]. See Additional file 3 for detailed bioinformatics methods.

### Metabolomics sample preparation and LC–MS/MS data acquisition

Within 4 months of collection 50–80 mg of digesta from each sample was thawed and weighed into 2 mL Qiagen homogenization tubes (catalog no. 990381). Qiagen stainless steel beads were added to each tube followed by 50% MeOH/H_2_O in a 1:20 w/v ratio. The samples were homogenized at 25 Hz for 5 min in a Qiagen TissueLyzer II (Qiagen, Hilden, Germany) then allowed to extract at 4 °C for 1 h. After this time, the samples were centrifuged at 14000 rpm for 15 min in an Eppendorf US centrifuge 5418 (USA) to pellet the cellular debris and 200uL of supernatant from each sample was transferred to a shallow polypropylene 96-well plate. The extracts were dried *in vacuo* using a Labconco Centrivap (USA), then sealed and stored at − 80 °C until LC–MS/MS analysis. Just prior to analysis, the extracts were reconstituted in 200uL of 50% MeOH/H_2_O solution containing 1uM sulfadimethoxine (CAS 122-11-2) as an internal standard.

LC–MS/MS analysis was performed on a Thermo UltiMate 3000 UPLC system coupled to an ultrahigh resolution quadrupole time of flight (qToF) mass spectrometer (Bruker Daltonics MaXis HD). A polar C18 column (Kinetex polar C18, 100 × 2.1 mm, 2.6 µm particle size, 100 A pore size—Phenomenex, Torrance, CA USA) was used for chromatographic separation. A high-pressure binary gradient pump was used to deliver the mobile phase, which consisted of solvent A (100% water + 0.1% formic acid) and solvent B (100% acetonitrile + 0.1% formic acid). The flow rate was set to 0.5 mL/min and the injection volume for each sample was 5uL. Following injection, samples were eluted with the following linear gradient: 0–1 min, 5% B; 1–9 min increasing from 5 to 100% B; 9–11 min, 100% B; 11–11.5 min decreasing from 100 to 5% B, 11.514 min, 5% B. Data collected after 11 min were excluded from analysis. All MS data were obtained using electrospray ionization (ESI) in positive mode, and the following settings were used: capillary voltage of 4500 V, nebulizer gas pressure of 2 bar, ion source temperature of 200 °C, and dry gas flow of 9 L/min. All spectra were collected using data dependent acquisition (DDA), where the spectral rate was set to 3 Hz and 10 Hz for MS1 and MS2, respectively. The five most intense ions per MS1 were selected for MS/MS acquisition and an active exclusion was enabled, which allowed two MS/MS spectra and was released after 30 s, at which point the precursor ion was reconsidered for MS/MS if the ratio of current intensity to previous intensity > 2. Lock mass calibration was then applied for the internal calibrant hexakis (1H,1H,2H-perfluoroethoxy) phosphazene (CAS 186817-57-2) and the raw data (.d) was converted to.mzXML format using Bruker DataAnalysis software. The data was pre-processed with MZMine2 (see Additional file 3 for parameters) and run through the GNPS feature-based molecular networking (FBMN) workflow [59, 60].

### Statistical analysis

All statistical analysis were performed in Rstudio (Version 1.2.5033). The curated microbial 16S rRNA gene data were imported into Rstudio and the effect of location within the gut was tested for multivariate diversity metrics and univariate ASV relative abundances. Alpha diversity metrics sobs (observed ASVs), Shannon evenness, and Shannon–Weaver [61] were found to be normally distributed and were run in mixed models (lmer function) using gut subsection as a categorical variable and fish individual ID as a random effect. By assigning each fish a random effect value in a mixed model, we were able to account for the non-independence of different gut subsections within a given fish prior to assessing the significance of gut subsection. All subsequent univariate models were run using the same mixed model structure. Effect significance was analyzed using a Type III Analysis of Variance (anova function, ddf = Kenward-Roger) and pairwise comparisons between gut subsections were done using the lsmeans function with adjust = “tukey”. Multivariate community structure differences were tested using weighted Unifrac distances and PERMANOVA (adonis2 and pairwise.adonis functions) with gut subsection as a categorical predictor variable using marginal sum-of-squares testing [62, 63]. Microbial community dispersion was calculated (betadisper function) from weighted Unifrac distances and square root transformed to fit a Guassian distribution. Transformed dispersion values were run in a mixed model and effect significance was analyzed in the same manner as alpha diversity.

To elucidate longitudinal shifts in ASVs associated with algal digestion, the dataset was narrowed to only midgut and hindgut samples. The stomach is physically distinct (Fig. 1) from the rest of the mid/hindgut and therefore was excluded from longitudinal analyses so as not to obscure trends in continuous spatial differentiation of ASVs during the digestive process. A subset of widespread and/or abundant ASVs were tested for longitudinal shifts: those with relative abundance ≥ 0.0005 in three or more samples or relative abundance ≥ 0.01 in one or more samples, which comprised a subset of 749 ASVs. These ASVs were arcsine-sqrt transformed to fit a Gaussian distribution and subsequently modeled and tested using the aforementioned mixed model structure and significance testing. P-values were corrected for multiple comparisons with the p.adjust function using the Benjamini–Hochberg method [64].

Extracted ion chromatogram (XIC) peak area data generated with MZmine2 was used for metabolomic analysis. Metabolomic ion features were defined as contaminants and removed from the dataset if the average XIC value in all samples for a given metabolite was less than or equal to twice the maximum XIC value of said ion feature in the blanks. Any low abundance ion feature that appeared in 3 or fewer samples was also removed from the dataset, yielding a total of 1133 metabolites for downstream analysis. Ion feature XIC values were relativized to total sample XIC to generate metabolite relative abundances and used to generate a Bray–Curtis dissimilarity matrix. PERMANOVA and dispersion analysis were performed on the Bray–Curtis dissimilarity matrix in the same manner as the 16S rRNA gene data. Dispersion data was normal and thus was not transformed prior to running mixed models.

In parallel to the ASV data, we selected only GI and HG samples to elucidate longitudinal shifts in metabolites associated with digestive processes beyond the stomach with similar thresholding yielding 549 metabolites for longitudinal testing. These metabolites were arcsine-sqrt transformed to fit a Gaussian distribution and subsequently modeled and tested using the same approach as the ASV data.

Microbiota and metabolome multivariate correlation was investigated using a Mantel test between the 16S rRNA gene unifrac distance matrix and the metabolomic Bray–Curtis dissimilarity matrix [65]. An additional Procrustes test was performed to confirm and visualize microbiota-metabolome correlation.

## Results

### Microbial diversity

We used 16S rRNA gene amplicon sequencing to assess shifts in the microbiota of the nenue gut as it was transected from stomach to hindgut. DNA from 37 fish gut samples were successfully sequenced, quality controlled, and subsampled. This yielded a total of 4 stomach samples (“ST”), 18 midgut samples spanning four subsections (“GI 1–4”), and 15 hindgut samples spanning three subsections (“HG 1–3”) that were subject to downstream analysis. The curated 16S rRNA gene dataset contained a total of 3866 amplicon sequence variants (ASVs). The number of observed bacterial ASVs differed significantly between gut subsections (F = 13.3, p = 4.3e−07; Additional file 5, Additional file 6a). The highest richness was found in ST (552 ∓ 85 ASVs) and lowest in GI 2 (135 ∓ 14 ASVs), with observed ASVs progressively increasing from GI 2 to HG 1–3 (Additional file 5, Additional file 6a). Microbial community evenness (Shannon evenness) also differed significantly between gut subsections (F = 7.1, p = 9.9e−05), increasing continuously from ST (0.46 ∓ 0.10) to HG 3 (0.77 ∓ 0.03) (Additional file 5, Additional file 6b). Lastly, the composite evenness and richness Shannon–Weaver diversity metric was significantly lower in ST and early GI compared to the HG (F = 10.2, p = 5.2e−06, Additional file 5, Additional file 6c).

### Microbiota multivariate analysis

**Fig. 2:**
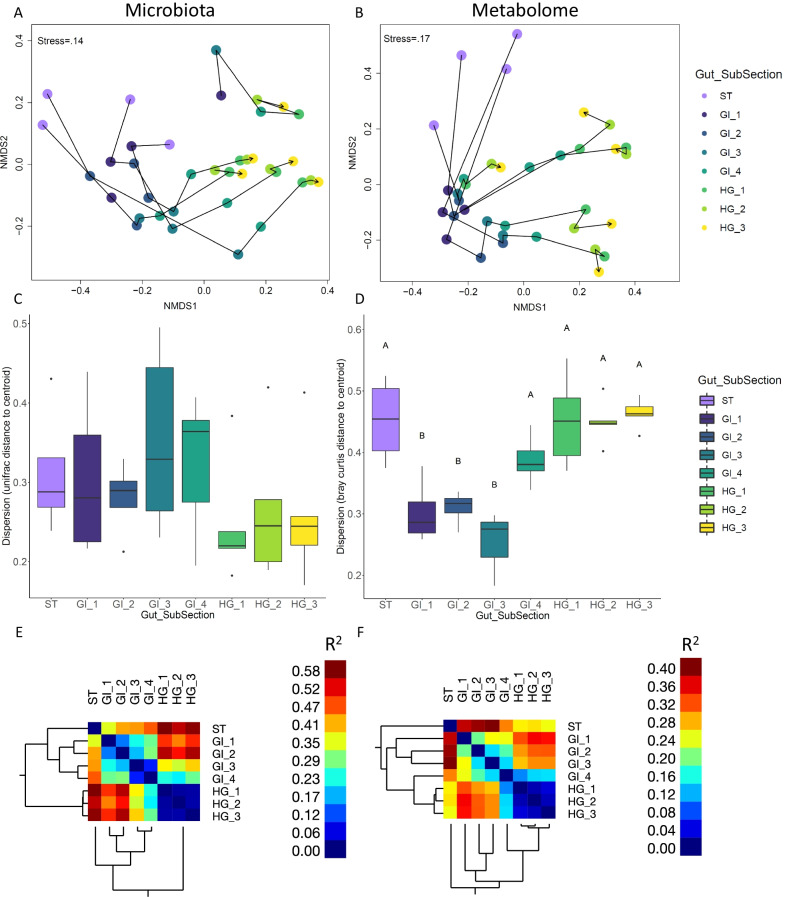
Microbial (**A**, **C**, **E**) and metabolomic (**B**, **D**, **F**) multivariate analysis of nenue gut subsections. Non metric multidimensional scaling plots of the weighted unifrac distances among microbial communities (**A**) and bray Curtis distances among metabolomic samples (**B**) from the nenue gut. Samples are colored by gut subsection. Arrows track the transition of microbiota/metabolomes in an individual fish, starting in the stomach and ending in the hindgut. Box and whisker plots depict multivariate dispersion of microbial communities (unifrac distance to centroid) (**C**) and metabolomic samples (bray curtis distance to centroid) (**D**). Samples are color coded by gut subsection. Dispersion in both microbial communities and metabolomic samples responded significantly to gut subsection, but only metabolomic samples showed significant post hoc pairwise differences (α = .05, Tukey Post Hoc test), which are indicated by letter significance indicators. Clustering heatmaps depict pairwise R^2^ values between samples from PERMANOVAs for (**E**) microbiota and (**F**) metabolome datasets

Microbial community structure was significantly different between the broad categories of gut section (PERMANOVA, R^2^ = 0.45, p < 0.001). Community structure also differed significantly at the finer spatial scale of gut subsection (PERMANOVA, R^2^ = 0.49, p < 0.001) (Fig. 2a; Table 1). Microbial community dispersion varied significantly across gut subsections (F = 2.6, p = 0.037), with communities increasing in dispersion as they progressed from ST to the end of GI and then becoming homogenized (lower dispersion) in all three HG samples (Fig. 2c, Additional file 7).

**Table 1:** PERMANOVA results for microbial communities and metabolomes tested against gut subsection

	R2	F	Pr(> F)
Gut subsection (microbial)	0.49	3.9901	0.001
Gut subsection (metabolomes)	0.35	2.0834	0.001

Trends in the NMDS ordination and pairwise PERMANOVA indicated a continuous transition of microbial communities throughout the gut (Fig. 2a, e; Additional file 8), with communities starting in ST and progressively differentiating through the GI subsections 1–4 and into HG 1–3. Pairwise PERMANOVA testing showed four significantly different gut regions, “Stomach” (ST), “Early-Midgut” (GI 1 and GI 2), “Late-Midgut” (GI 3 and GI 4), and “Hindgut” (HG 1, HG 2, and HG 3). R^2^ data derived from the pairwise PERMANOVA, which indicates levels of pairwise variation between gut subsections, further supports this conclusion, showing that microbial communities varied substantially between regions and remained similar within a given region (Fig. 2e; Additional file 8).

**Fig. 3:**
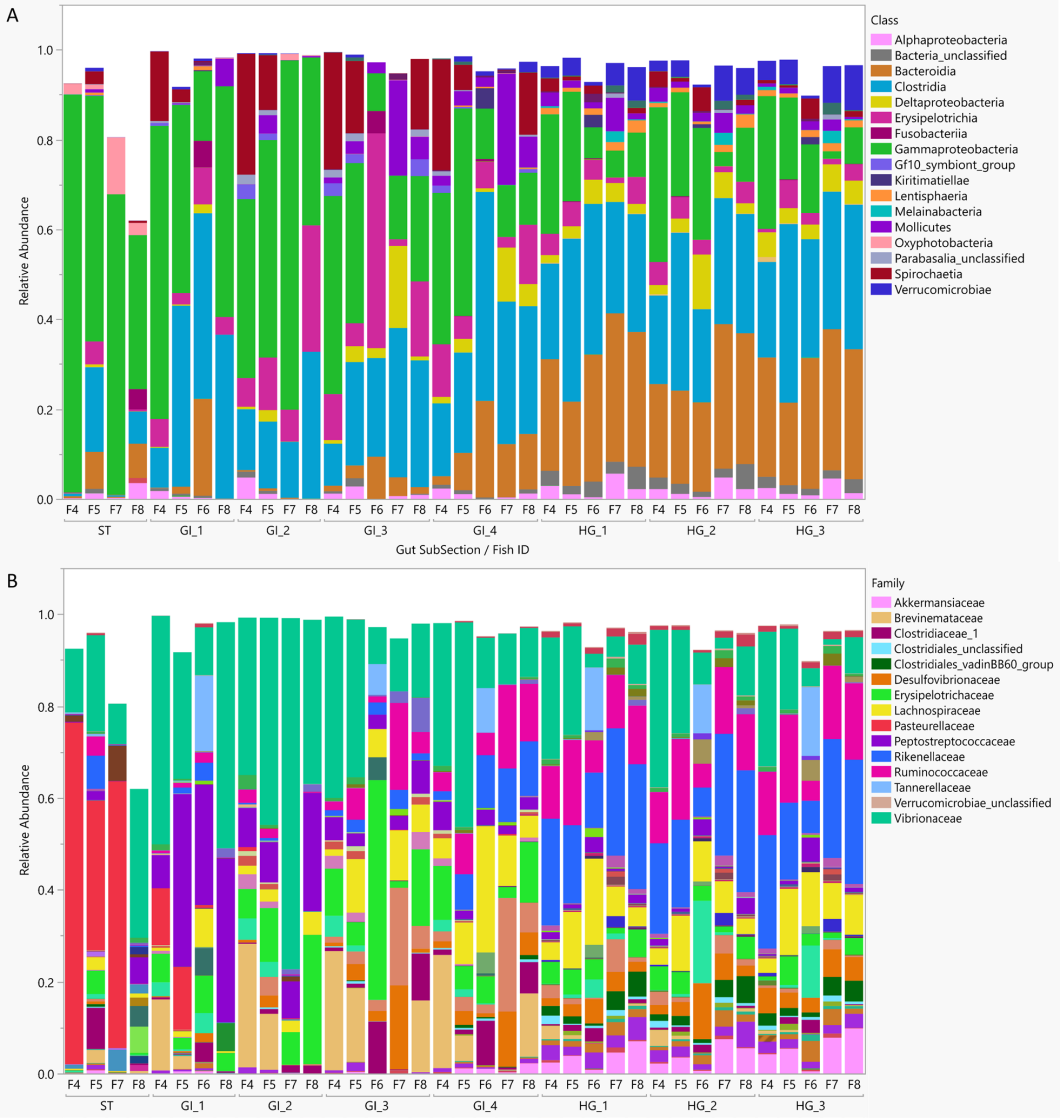
Stacked bar chart of the average relative abundances of common ASVs represented at the Class level (**A**) and Family level (**B**) in the nenue gut subsections. ASVs were considered “common” if their relative abundance ≥ .0005 in samples ≥ 3 or relative abundance ≥ .01 in samples ≥ 1

All gut samples were dominated by the classes *Gammaproteobacteria* (31.5%), *Clostridia* (24.5%), *Bacteroidia* (12.8%), and *Erysipelotrichia* (6.8%) (Fig. 3a). Microbial families demonstrated strong spatial differentiation across the nenue gastrointestinal tract (Fig. 3b): *Vibrionaceae* was common throughout the nenue gut (peaking at a relative abundance of 48% in GI 2), stomach samples were dominated by *Pasteurellaceae* (39%), followed by a sequence of families belonging to various anaerobic bacterial classes (primarily *Clostridia* and *Bacteroidia*) from GI 1 to HG 3. *Peptostreptococcaceae* peaked in GI 1 (27%), *Erysipelotrichaceae* peaked in GI 2 and GI 3 (13% and 16%, respectively), *Lachnospiraceae* peaked in GI 4 (11%), and HG 1–3 were dominated by *Rikenellaceae* (21%, 20%, and 20%, respectively) and *Ruminococcaceae* (12%, 12%, and 14%, respectively) (Fig. 3b).

### Metabolomic multivariate analysis

Although untargeted metabolomics is generally unable to resolve dynamics of small, polar molecules such as SCFAs, this approach paints a better picture of the entire chemical microenvironment and resolves changes in other key chemical classes that we know little about in fish guts. Shifts in untargeted metabolome multivariate structure along the nenue gut paralleled that of the microbial communities. Metabolomes differed significantly by the broad categories of gut section (PERMANOVA, R^2^ = 0.26, p < 0.001) as well as the finer spatial scale of gut subsection (PERMANOVA, R^2^ = 0.35, p < 0.001) (Fig. 2b; Table 1). Metabolome dispersion exhibited the opposite trend of microbial community dispersion, varying significantly across the gut subsections (F = 16.5, p = 1.3e−07), with the highest dispersion in the ST, GI 4, and HG 1–3, and lowest dispersion in GI 1–3 (Fig. 2d, Additional file 7).

Gut metabolomes and microbiota tightly “tracked” each other as they shifted through the nenue gut. Generally, metabolomes exhibited spatial trends that were similar to those seen in the microbial communities (Fig. 2b; Additional file 9), with metabolomes continuously differentiating from the ST, though GI 1–4, and into the HG (HG 1–3). Gut microbiota and metabolomes exhibited significant multivariate correlation (Mantel test, r = 0.4342, p = 0.001) and showed similar patterns in ordination space (Procrustes test, sum of squares = 0.7124, p = 0.001), further indicating the parallel shifts of these two sample types and the potential feedback between microbial communities and metabolomes in this system (Additional file 10).

Pairwise PERMANOVA results for the metabolomes broadly showed similar trends as the microbial community data: metabolomes differentiated progressively from ST to HG 3 and the HG section (HG 1–3) exhibited uniform metabolome structure (Fig. 2f; Additional file 9). Despite this, there were no significant pairwise differences in metabolomes between each gut subsection, likely due to the relatively small sample size prior to p-value adjustment (Additional file 9). There were other notable differences between microbial communities and metabolomes as well. Namely, where microbial communities showed clear and significant clustering of gut subsections into 2 regions in the midgut (“Early-Midgut” and “Late-Midgut”), R^2^ values derived from pairwise PERMANOVA of the metabolomes showed no such clustering and instead exhibited a continuous gradient of differentiation within the midgut (Fig. 2f, Additional file 8, Additional file 9). Additionally, while GI 4 is distinct from the HG in the microbial communities, in the metabolomes it appears to be more similar to the HG than the rest of the GI (Fig. 2f, Additional file 8, Additional file 9).

### Differential ASV analysis

**Fig. 4:**
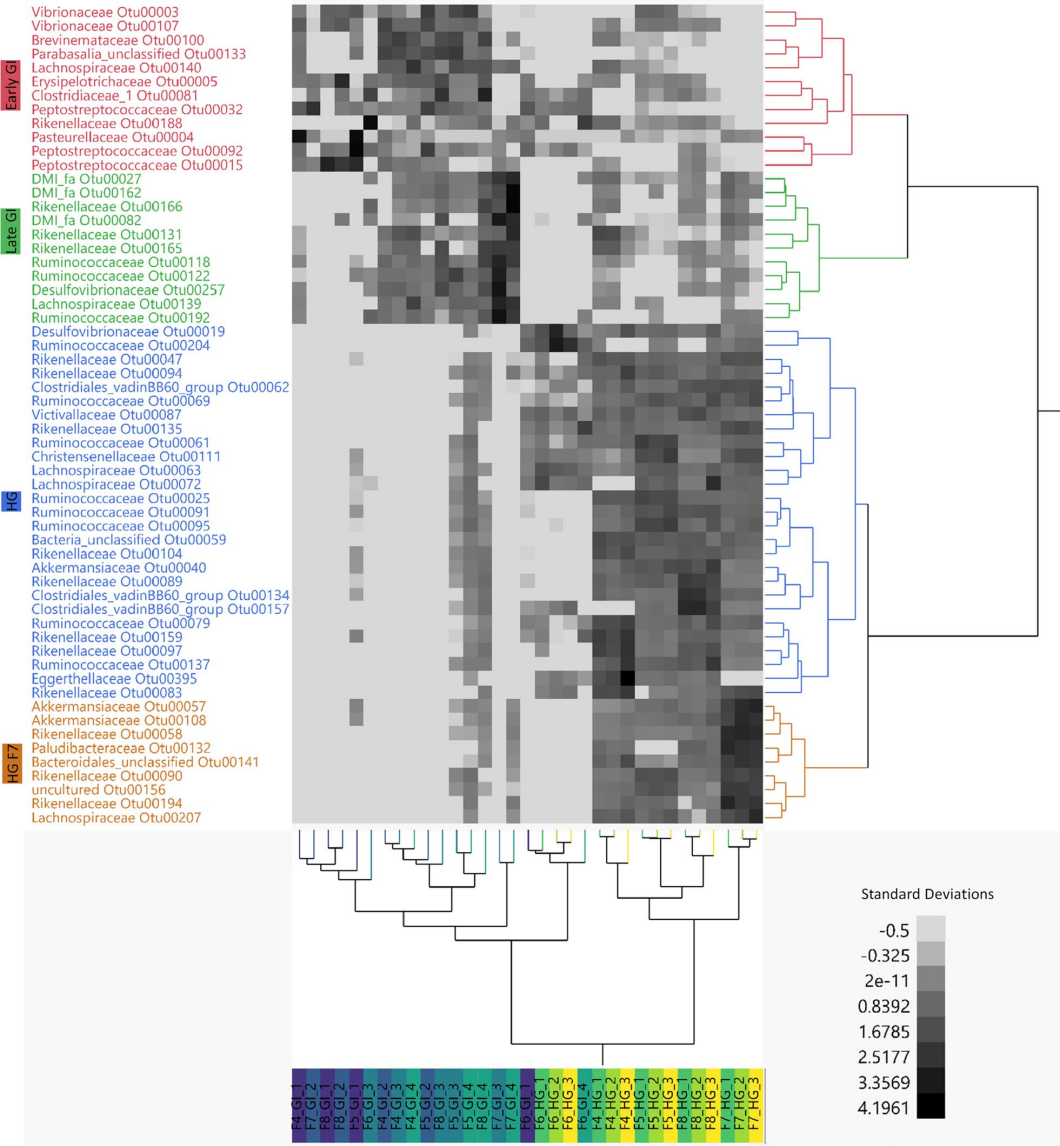
ASV enrichment between samples of the 58 most abundant ASVs that responded significantly to gut subsection (adjusted p-value ≥ .05). ASV relative abundances were arcsine squareroot transformed and z-scored to generate a two-way heatmap. Samples are clustered on the x axis and ASVs are clustered on the y axis. ASVs are broadly clustered into 4 modules of enrichment (CCC = 7.7245) corresponding to “Early GI,” “Late GI,” “HG,” and “HG F7.” ASVs are colored according to these clusters and samples on the x axis are color coded according to gut subsection

**Fig. 5:**
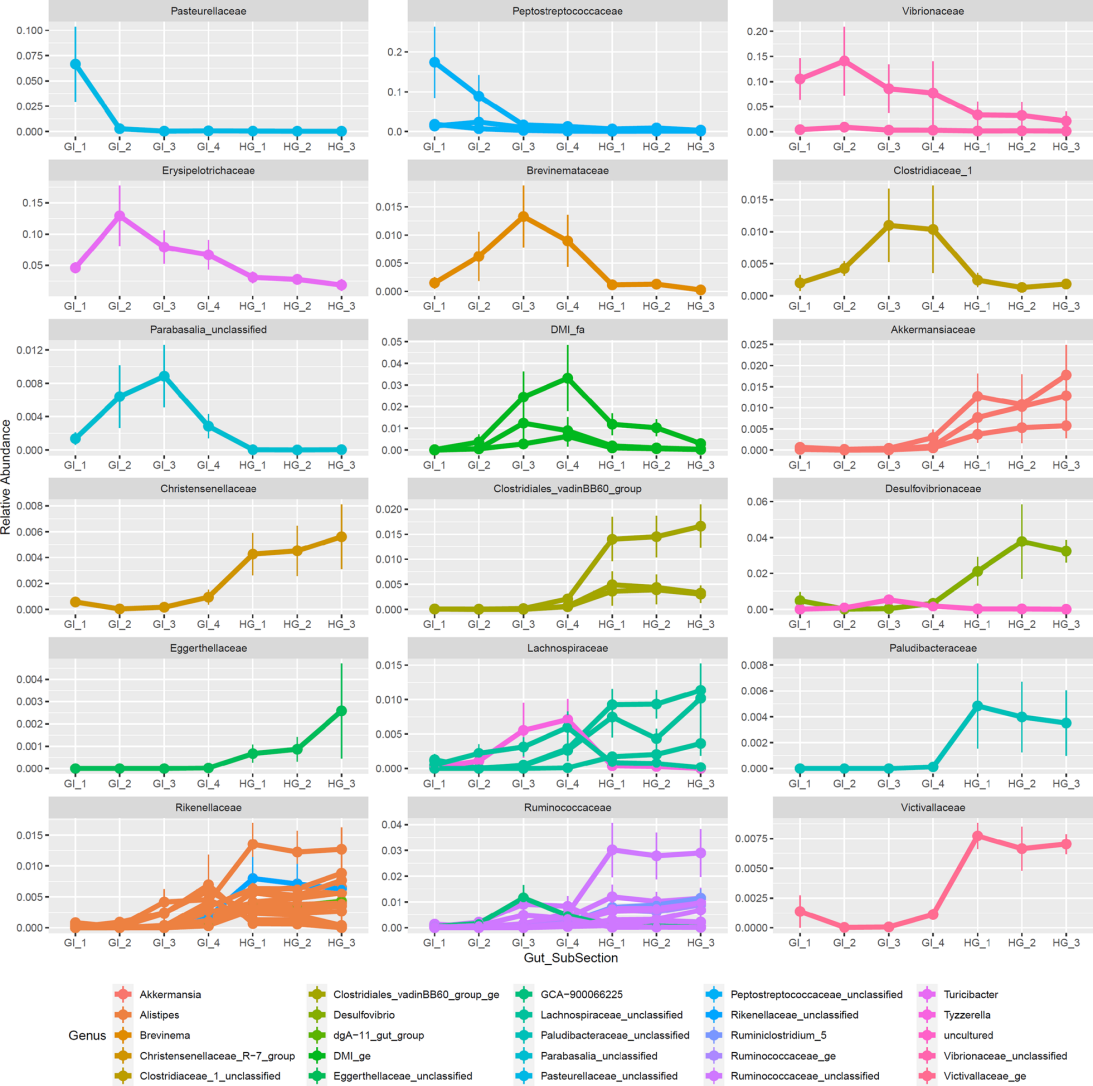
Family-level trends across the Nenue gut in the 58 most abundant ASVs that responded significantly to gut subsection (adjusted p-value ≥ .05). Average ASV relative abundance is plotted against gut subsection, with plots separated by Bacterial Family, ASVs colored by Genus, and vertical lines indicating standard error. Plots are ordered by gut subsection enrichment from anterior to posterior

To elucidate spatial changes in ASVs associated with digestive processes beyond the stomach, the dataset was narrowed to only GI and HG samples and mixed models were run on arcsine squareroot transformed relative abundance data of the 749 most abundant ASVs in these samples. A total of 283 ASVs responded significantly to gut subsection (p ≤ 0.05 after Benjamini–Hochberg p-value correction for multiple comparisons) (Additional file 11). To identify only the most abundant of these significant ASVs, we further selected ASVs that had a relative abundance ≥ 10% in at least one sample, which yielded a total of 58 high abundance ASVs that responded significantly to gut subsection (Figs. 4, 5; Additional file 12).

We used two-way hierarchical clustering of z-scored arcsine squareroot transformed relative abundance data to visualize the 58 most abundant ASVs that responded significantly to gut subsection. Significant ASVs clustered into four broad modules (cubic clustering criterion: 7.7245) corresponding to GI 1–2 enriched ASVs (“Early-GI”), GI 3–4 enriched ASVs (“Late-GI”), HG enriched ASVs (“HG”), and HG enriched ASVs particular to Fish 7 (“HG F7”) (Fig. 4; Additional file 12). The majority (36/58, 62%) of ASVs were enriched in the HG (either “HG” cluster or “HG F7” cluster), 19% (11/58) were enriched in the Early-GI and 19% (11/58) were enriched in the Late-GI (Fig. 4; Additional file 12).

The trends in significant ASV analysis corroborated our family level observations, with ASVs from different families generally demonstrating consistent spatial differentiation patterns (Fig. 5). *Pasteurellaceae* and *Peptostreptococcaceae* ASVs peaked in relative abundance at GI 1, *Vibrionaceae* and *Erysipelotrichaceae* ASVs peaked in relative abundance at GI 2, *Brevinemataceae*, *Clostridiaceae*, and ASVs in the class *Parabasalia* peaked at GI 3, and certain ASVs in the class *Mollicutes*, the family *Lachnospiraceae*, and the family *Rikenellaceae* peaked at GI 4. Numerous families of ASVs peaked in relative abundance in the HG including *Desulfovibrionaceae*, *Akkermansciaceae*, and *Lachnospiraceae*. HG samples also had higher relative abundances of ASVs from common anaerobic, gut-associated families *Rikenellaceae* and *Ruminococcaceae*. These two families contained the most ASVs that responded significantly to gut subsection (15 *Rikenellaceae* ASVs, 25% of total; 11 *Ruminococcaceae* ASVs, 19% of total).

### Differential metabolite analysis

**Fig. 6:**
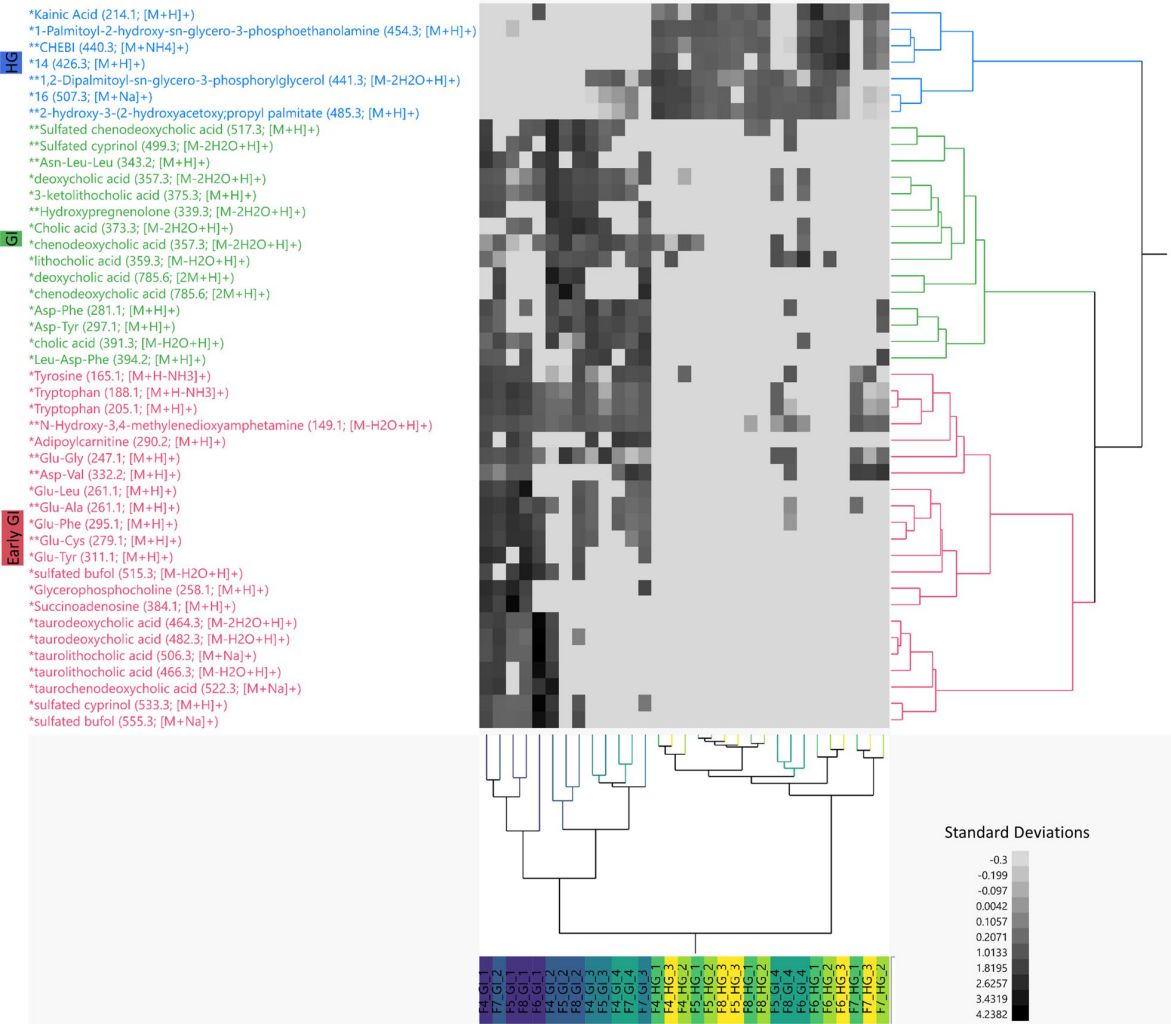
Enrichment between samples of the 44 most abundant metabolites that responded significantly to gut subsection (adjusted p-value ≥ .05) and had known library or analog IDs. Metabolite relative abundances were arcsine squareroot transformed and z-scored to generate a two-way heatmap. Samples are clustered on the x axis and metabolites are clustered on the y axis. Metabolites are labeled by their library and/or analog ID, with one asterisks indicating a match class level 2 (library ID) and two asterisks indicating a match class level 3 (analog ID). M/z values and molecular adducts are indicated in parenthesis after each feature name. Metabolites are broadly clustered into 3 modules of enrichment (CCC = 7.072) corresponding to “Early GI,” “GI,” and “HG.” Metabolites are colored according to these clusters and samples on the x axis are color coded according to gut subsection

**Fig. 7:**
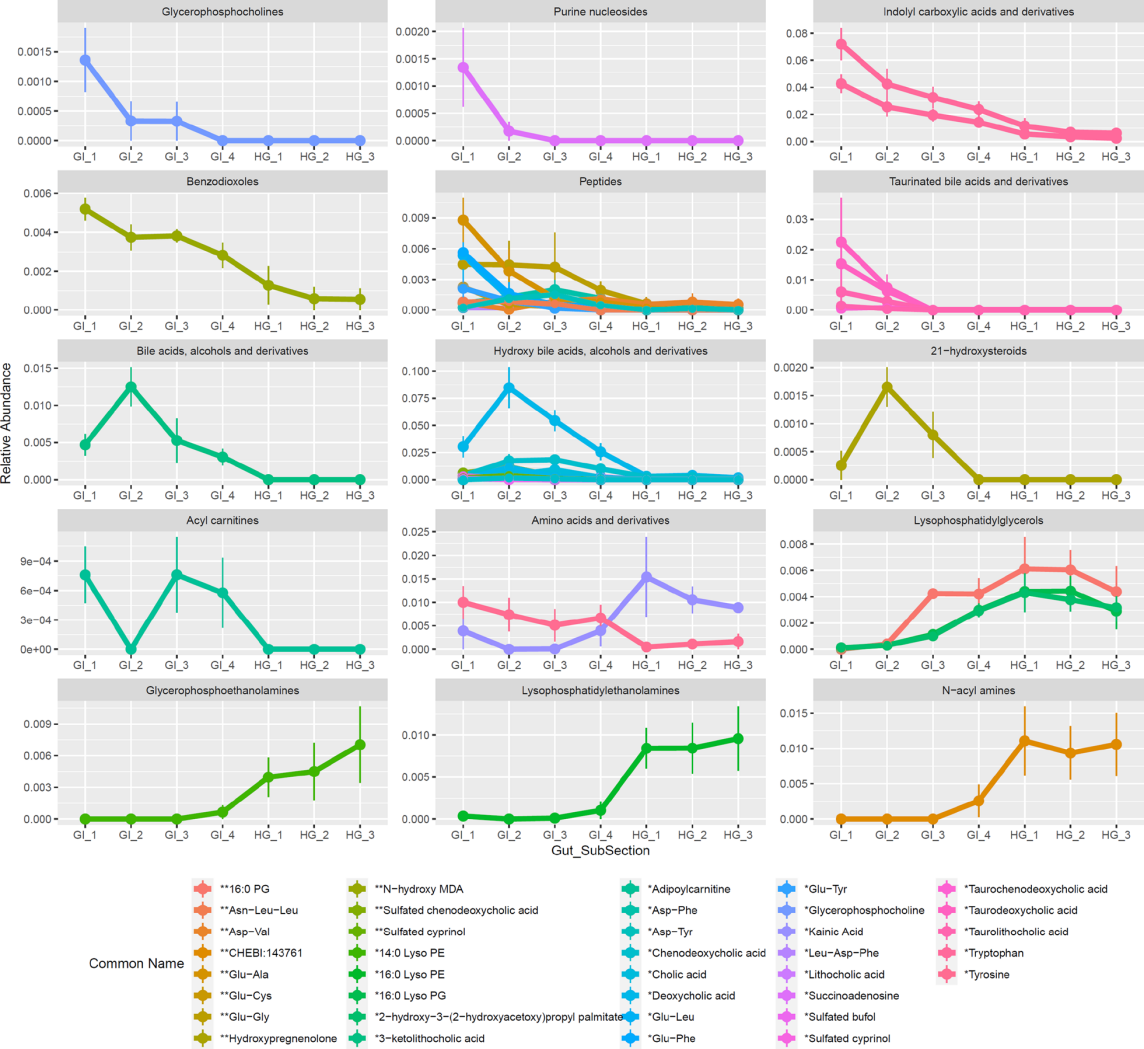
ClassyFire Parent Level 1 trends across the Nenue gut in the 44 most abundant metabolites that responded significantly to gut subsection (adjusted p-value ≥ .05) and had known library or analog IDs. Average metabolite relative abundance is plotted against gut subsection, with plots separated by ClassyFire Parent Level 1, metabolites colored by library and/or analog ID, and vertical lines indicating standard error. Metabolites are labeled by their common library and/or analog ID, with one asterisks indicating a match class level 2 (library ID) and two asterisks indicating a match class level 3 (analog ID). Plots are ordered by gut subsection enrichment from anterior to posterior

In parallel to the differential ASV analysis, we investigated the spatial changes of the top 549 most abundant metabolites in the GI and HG. We found 64 metabolites that varied significantly between gut subsections (FDR-adjusted p ≤ 0.05) (Additional file 13). To further identify metabolic processes occurring along the nenue gut tract, the 64 metabolites were reduced to only include metabolite features with exact or analog spectral matches to a known compound in GNPS which includes third-party libraries such as NIST, Metlin and Massbank, yielding a total of 44 metabolites with known chemical identities [60]. All metabolites that directly matched with GNPS library MS/MS spectra were level 2 classifications whereas those identified as analogs of library compounds were level 3 classifications according to the guidelines proposed by the Metabolomics Standards Initiative (MSI) [66]. We again used two-way hierarchical clustering of z-scored arcsine square root transformed relative abundance data to visualize these 44 metabolites that responded significantly to gut subsection. These 44 metabolites clustered into three broad modules (cubic clustering criterion: 7.1252) corresponding to GI 1–2 enriched metabolites (“Early-GI”), metabolites enriched broadly across the GI section (“GI”), and hindgut enriched metabolites (“HG”) (Fig. 6; Additional file 13). Of the 44 significant metabolites, 50% (22/44) were enriched in the Early-GI, 34.1% (15/44) were enriched in the GI and 15.9% (7/44) were enriched in the HG (Fig. 6; Additional file 13). Early GI enriched metabolites included glycerophosphocholine, succinoadenosine, tryptophan, and glutamate dipeptides (Figs. 6, 7). Bile acids were relatively abundant in the Early GI and bile acid deconjugation was observed. Specifically, taurine conjugated bile acids peaked in GI 1 and then rapidly declined in abundance, followed by unconjugated bile acids and unconjugated hydroxy bile acids which peaked in GI 2 (Fig. 7). Metabolites enriched broadly across GI (but not in HG) included certain aspartate dipeptides and tyrosine. Lastly, the HG was enriched primarily in phospholipids.

## Discussion

Microbes are found in ubiquitous association with multicellular organisms and perform numerous functions for the host. This holds especially true for gastrointestinal symbiosis; in humans and other terrestrial organisms, microbial communities encode diverse metabolic functions to digest complex nutritional resources, producing essential metabolites for both themselves and their hosts [7, 67]. Marine herbivorous fish play important ecological roles in coastal systems by consuming benthic algae and thus maintaining balanced benthic community structure [15, 16]. However, little is known about how these fish digest algae and how gut microbial communities might assist in this process, which is a priority for piscine herbivory research [11]. Specifically, no studies have examined paired microbial and chemical changes along the gastrointestinal tract at high spatial resolution and what this might indicate about the catabolic processes contributing to algae decomposition occurring in the gut. In order to address these knowledge gaps, we applied a parallel 16S rRNA gene amplicon sequencing, tandem LC–MS/MS approach to profile spatial changes in microbial communities and metabolite pools and what this might imply about algal deconstruction in nenue. We opted not to focus on microbiota/metabolome differences between fish species, life stages, etc., and instead specifically attempted to evaluate the consistency of longitudinal variation across the gut microbiome of a representative set of Kyphosids. We found high degrees of correlation between microbiota and metabolomes, previously uncharacterized variability in these microbe/metabolite pools within the midgut, and parallel changes in microbes and metabolites that reveal patterns of gut metabolism by dominant *Clostridia* and *Bacteroidia* classes of Bacteria.

### Multivariate correlation between microbiota and metabolomes

Broadly, our study suggests that both microbial communities and metabolomes are structured according to gut subsection (Fig. 2a, b; Table 1). Here, microbial communities and metabolomes demonstrated parallel spatial differentiation as they transitioned from the anterior end (stomach) to the posterior end (hindgut) of the gastrointestinal tract. Multivariate analysis including NMDS ordinations, Mantel tests, and Procrustes tests/visualizations show that microbial communities and metabolomes consistently transition in the same manner from the stomach, through the midgut, and into the hindgut (Fig. 2a. b).

Pairwise PERMANOVAs revealed high levels of microbiota variation within the midgut (GI). Microbial community structure differentiated into four distinct gut regions, Stomach (ST), Early-Midgut (GI 1–2), Late-Midgut (GI 3–4), and Hindgut (HG 1–3) (Fig. 2e; Additional file 8) with most of the spatial differentiation in microbial communities occurring in the stomach and midgut, while all microbial communities were homogeneous within the hindgut. This is in accordance with the morphology of the nenue guts we sampled; the distance between the stomach and posterior end of the midgut was much longer than the length of the hindgut, thus allowing for longer transit time and spatial area for microbial communities to differentiate (Additional file 1).

Metabolomes exhibited similar spatial differentiation patterns to microbial communities, with metabolomes transitioning from the stomach through the midgut, and ending in the hindgut, which again maintained uniform metabolomic structure (Fig. 2f). While microbiota showed clear clustering of communities in the midgut, the metabolomes showed more continuous differentiation. One potential explanation is that metabolomes are being continually transformed via microbial activity through the midgut, while the same microbial community in a given gut region can perform multiple processes. Thus, microbial communities can remain stable while changing activities, resulting in gradients of metabolomes that do not show the same discrete clustering as the corresponding microbial communities. In both the microbiota and metabolomes, our fine-scale spatial sampling identified previously uncharacterized variation in the midgut, indicating microbiota and chemical differentiation within this morphologically uniform region that is often not captured in current sampling designs.

Multivariate dispersion responded significantly to gut subsection in both microbial communities and metabolomes, although the nature of the response differed. Microbial community dispersion decreased as the gut was transected from anterior to posterior (Fig. 2c) which has also been observed in other marine herbivorous fishes including rabbitfish [29, 31]. This suggests that gut microbiota converge on a uniform community structure in the hindgut regardless of other factors, namely individual fish genotype and diet. Factors such as individual host genotype and diet often exert influence on fish gut microbial community structure, and fish of the family Kyphosidae are known to have diverse diets and have even been documented to be omnivorous on occasion [12, 38, 68–73]. The high beta diversity of gut microbiota in the early gut subsections could reflect the diversity of dietary contents between individual fishes. The subsequent convergence of microbial communities in the hindgut would then reflect both a homogenization of the diet derived chemical environment that feeds the gut microbiota and a concurrent homogenization of the gut microbiota to a specific community that serves a specific metabolic function. This possibility is corroborated by a study on the gut microbiota of Grass Carp fed two unique diets, in which midgut microbial communities were highly sensitive to diet whereas hindgut microbial communities were not [20]. This could mechanistically explain how individual diet variation between fish leads to high beta diversity in the midgut and lower beta diversity in the hindgut.

The metabolomic data, however, show the opposite trend in dispersion, with the most variable metabolomes in the stomach and hindgut and the most homogeneous metabolomes in the midgut (Fig. 2d). This contradicts the hypothesis that variation in gut chemistry drives variation in gut microbial communities. Instead, we find that the most variable microbial communities correspond to the most homogeneous metabolomes. Fish are known to release an array of small molecules such as bile acids into the early portions of their midgut to aid digestion; it is possible that these fish-derived metabolites are so abundant in the nenue midgut that they homogenize the metabolomes in the midgut relative to the hindgut [74]. Bile acids indeed made up a large portion of the metabolome in the early midgut samples, which could very well be driving the observed trends in dispersion (Figs. 6, 7).

### Microbial ASV changes across the nenue gut

Microbiota species richness and evenness shifted through the gastrointestinal tract (Additional file 5, Additional file 6); species richness was highest in the stomach, lowest immediately after in the early midgut, and then increased in the late midgut until reaching a peak in the hindgut. Species evenness increased continuously from anterior to posterior. This general increase in species richness and evenness through the nenue gut corroborates results from other studies on marine fishes [31, 75]. Jones et al. 2018 observed highest alpha diversity in the hindgut in the rabbitfish *Siganus fuscescens* while Gajardo et al. 2016 found similar trends in the taxonomically and geographically distinct Atlantic Salmon *Salmo salar* [31, 75]. However, the opposite pattern has also been found, suggesting that alpha diversity trends through the gut may vary by other factors besides gut region [23, 24, 29, 30].

Many of the differentially abundant ASVs shifting through the nenue gut are in the order *Clostridiales* and are commonly found in other herbivore guts, both terrestrial and marine [43, 69]. These include *Peptostreptococcaceae* (genus: unclassified, ASVs 15, 32, and 92), *Vibrionaceae* (genus: unclassified, ASVs 3 and 107), *Brevinemataceae* (genus: *Brevinema*, ASV 100), and *Erysipelotrichaceae* (genus: *Turicibacter*, ASV 5) ASVs in the Early-GI (GI 1 and GI 2), and *Lachnospiraceae* (genus: *Tyzerella*, ASV 139) and *Rikenellaceae* (genus: *Alistipes*, ASVs 131, 165, and 166) in the Late-GI (GI 3 and GI 4). Many ASVs significantly enriched in the early GI belong to known obligate anaerobic fermenting bacterial families including *Peptostreptococcaceae* and *Erysipelotrichaceae* (genus: *Turicibacter*) and have been identified as putative gut commensals in a variety of organisms including humans, pigs, rats, and other marine herbivores such as rabbitfish, tilapia, and parrotfish [14, 17, 29, 30, 76–82]. The high relative abundance of these ASVs early in the midgut suggests that the nenue gut is anaerobic much sooner than expected, and anaerobic breakdown of algal biomass is already occurring immediately posterior to the stomach. The late midgut subsections, especially GI 4, begin to more closely resemble the hindgut, with some ASVs from two abundant HG families, *Lachnospiraceae* and *Rikenellaceae*, peaking in relative abundance in this section. Previously observed metabolic similarities between the late midgut and hindgut concur with these microbiota observations, indicating that both microbial communities and metabolism in the late midgut begin to resemble that of the hindgut [39].

The hindgut was enriched predominantly in ASVs from families *Ruminococcaceae* (ASVs 25, 61, 69, 79, 91, 95, 137, and 204), *Lachnospiraceae* (ASVs 63, 72, and 207), and *Rikenellaceae* (ASVs 47, 58, 83, 90, 94, 97, 104, 135, 159, and 194), and generally resembled the microbial community structure of other herbivores that have anaerobic, fermentative hindguts. ASVs from *Ruminococcaceae* and *Lachnospiraceae* remained largely unclassified at the genus level, whereas most *Rikenellaceae* ASVs were classified as *Alistipes*. *Alistipes* (along with the entire *Rikenellaceae* family) are obligate anaerobes and produce succinate as their metabolic end-product via fermentation [83–85]. This genus is commonly found in the alimentary tract of animals including humans and fish (specifically members of the Kyphosidae family) [21, 34, 86–88]. These lines of evidence strongly suggest that *Alistipes* play a major role in hindgut metabolism of healthy nenue. *Ruminococcoceae* and *Lachnospiraceae* are both common gut endosymbionts and important in anaerobic digestion of plant compounds including ruminant cellulolytic digestions [89]. These two families are some of the most abundant in gut environments and contain a diverse array of fibrolytic enzymes [90–93]. Taken together, our nenue microbiota reveal novel microbial diversity in the midgut and strongly support the claim that terrestrial and marine herbivores are united in their use of anaerobic, fermentative bacterial gut endosymbionts to digest plant matter [32].

### Metabolite changes across the nenue gut

The chemical composition of the Kyphosid gut varies significantly across the gastrointestinal tract, largely due to changes in the relative abundance of a variety of compound classes essential for host metabolism: bile acids and alcohols, small peptides and amino acid-containing compounds, and phospholipids [94–96]. Bile acids were enriched in the Early-GI (GI 1 and GI 2), while small peptides and amino acids were found in high relative abundances across the entire GI (GI 1–4), and both play critical roles in either food breakdown or absorption, respectively. Host-derived or primary bile acids and alcohols are secreted into the small intestine of fish and other vertebrates to break up fats and lipids, but most of these are reabsorbed and recycled via enterohepatic circulation before passing to the hindgut [97]. The majority of primary bile acids and alcohols enter the fish gastrointestinal tract as bile salts conjugated with taurine or esterified with sulfate, respectively [98]. However, gut microbes in the early midgut can quickly hydrolyze these conjugated bile acids using bile salt hydrolases (BSHs) to detoxify their structures and to produce free taurine for anaerobic energy production [99]. By studying the nenue gut metabolome on such a fine spatial scale, both host bile acid secretion, microbial bile acid deconjugation, and host reabsorption were able to be distinctly observed. Specifically, taurine-conjugated bile acids were most abundant in GI 1 where they are secreted by the host, then free bile acids and alcohols peaked in GI 2 where they are produced by microbial bile acid deconjugation, and finally, bile acids were largely undetected in the hindgut suggesting nearly complete reabsorption back into the bloodstream in the Late-GI.

The midgut is also where dietary proteins are hydrolyzed into small peptides (< 4 AAs) and free amino acids [100]. Accordingly, many di- and tri-peptides containing the non-essential amino acids glutamate (Glu) and aspartate (Asp) were enriched across the entire nenue midgut (GI 1–4). Glu and Asp are both important energy sources for fish and are also abundant AAs in seaweed providing its distinctive umami flavor [19, 101, 102]. While the effects of dietary Asp and Glu have not yet been studied in fish, dietary supplementation with either amino acid improved growth performance in piglets and reduced the animal’s susceptibility to mycotoxins [103].

Previous studies have suggested that the primary role of the Kyphosid hindgut is to degrade algal polysaccharides through microbial anaerobic fermentation to release SCFAs and other volatile compounds that benefit host nutrition [33]. Although GC–MS is required to detect volatile metabolites such as SCFAs, untargeted LC–MS/MS analysis revealed that several phospholipids, such as palmitic acid (i.e. C_16_), were detected and found to be enriched in the hindgut, which is consistent with results from a previous study examining lipid digestion in turbot fish [104]. This finding suggests that there might be greater lipolytic activity in the fish hindgut compared to the stomach or midgut, though it could also be an artifact of bacterial cell lysis during metabolite extraction. Regardless, inclusion of certain dietary phospholipids has been shown to support healthy growth and development for many fish species in the early stages of life [96]. Therefore, it is essential to understand how these compounds are produced and utilized in adult fish and whether their abundances correlate with a particular bacterial species or community.

### Functional implications

Although we have taken no direct functional measurements in this study, the coupling of microbial and metabolomic data sheds light on the putative function(s) of microbial communities within the nenue gut. Bile acid deconjugation in the early midgut and phospholipid accumulation in the hindgut are two examples of how paired microbial-metabolomic data can be leveraged to infer the function of specific microbial communities. As previously mentioned, there were high abundances of conjugated (taurinated) bile acids in GI 1, which were subsequently deconjugated via microbial metabolism to produce high relative abundances of unconjugated bile acids in GI 2 (Figs. 6, 7). This implicates bacterial ASVs that peaked in abundance in the Early-GI, namely *Peptostreptococcaceae* (genus: unclassified) and *Erysipelotrichaceae* (genus: *Turicibacter*), as potentially acting in bile acid deconjugation in the nenue gut (Figs. 4, 5). Bacteria in the class *Clostridia* and phylum *Firmicutes*, which contain *Peptostreptococcaceae* family and *Turicibacter* genus, respectively, have been shown to perform bile acid deconjugation via BSH genes in other host organisms [99]. It is therefore reasonable to posit that *Peptostreptococcaceae* and *Turicibacter* play a similar role in the nenue gut.

We also observed elevated levels of palmitic acid-containing phospholipids in the hindgut (Figs. 6, 7). Bacteria are known to chemically transform palmitic acid into numerous phospholipids which can then modify host metabolism [105]. Additionally, phospholipids are an important chemical class for the development of healthy fish [94]. It is therefore likely that production of phospholipids in the nenue hindgut serves an important biological function for the host, and elucidating which bacteria are involved in this process will help us better understand the composition and function of a healthy herbivorous fish gut microbiota. The suite of bacterial ASVs enriched in the hindgut, predominantly in the *Rikenellaceae* and *Ruminococcaceae* families, are potential players in this process (Figs. 4, 5). Indeed *Alistipes*, the most abundant *Rikenellaceae* genus in the nenue hindgut, has been correlated with elevated levels of palmitic acid in rats, making it a likely suspect in this system as well [106]. We do not intend to draw concrete conclusions about gut microbiota function from these data but hope that these two examples reveal how paired microbial-metabolomic data can complement each other to provide insights into the function of gut microbiota and yield specific, testable hypothesis for future studies.

The major constraint of this study is its emphasis on high within-fish longitudinal resolution but relatively low sample size of individual fishes. A product of the vicissitudes of our requirement that wild-caught fish be dissected anaerobically immediately after removal from the ocean, our sample size was 5 fish from the genus *Kyphosus*, all of which varied morphologically and taxonomically. Our goal was not to explicitly evaluate the degree of microbiota/metabolomic variation between individual *Kyphosus* fishes but rather to elucidate the consistency of spatial patterns across the gastrointestinal tract. Whenever possible, we used random effects in mixed models to deal with this issue by accounting for inter-individual variation prior to testing the effect of gut subsection. However, future studies aiming to assess the spatial variation of microbiota/metabolomes along the gastrointestinal tract should aim to sample larger numbers of individual fish, ideally all within the same species, life stage, sex, etc., to control for inter-individual variation. In addition, complementary studies should be designed to explicitly assess the differences in microbiota/metabolomes between *Kyphosus* species, life stages, sexes, etc.

## Conclusions

This study reveals a clear, fine scale spatial differentiation and tight association of gut microbial communities and metabolomes through a marine herbivorous fish gastrointestinal tract. We also identified previously unobserved variation in microbial communities and metabolomes within the nenue midgut. These results can inform future fish gut microbiome studies to gain a more complete understanding of the microbial ecology of fish gut endosymbionts and the processes they mediate. Future studies must sample at a fine enough spatial scale to capture the extent of variation in the fish gut microbial ecosystem, i.e. a minimum of four regions throughout the mid- and hindgut.

Individual ASVs that exhibited differentiation through the gut were predominately facultative or obligate anaerobic gut endosymbionts, indicating that the nenue gut becomes anaerobic and potentially fermentative immediately posterior to the stomach, much earlier than previously suggested [33]. The chemical environment in the nenue gut changed dramatically though various gut subsections, largely due to changes in the relative abundance of several essential metabolites including bile acids and alcohols, small peptides and amino acid-containing compounds, and phospholipids. Additional spatial surveys of small, more polar metabolites such as SCFAs not captured by LC–MS/MS would pair well with our current data and shed light on additional metabolic and energetic intermediates along the fish digestive tract.

Combined microbiota-metabolome surveys of the gut help develop a more holistic understanding of the gut environment and the feedback between gut microbial communities and gut metabolomes. The significant covariation between gut microbial communities and metabolomes suggests there is likely strong feedback between microbes and metabolites in this system. Lastly, this study serves as a springboard from which functional hypothesis regarding herbivorous fish gut microbiota can be developed. We cannot in this manuscript describe functional relationships between bacteria and metabolites, or what metabolites are the substrates and/or products of specific bacterial metabolism. However, simple correlations between microbial communities and metabolomes can yield detailed functional hypothesis which can be further tested, such as deconjugation of bile acids by bacteria in the early midgut.

Future studies are necessary to address the complex interaction between microbial communities and metabolomes in relation to the digestion of marine algae. Specifically, enhanced experimental, computational, and statistical methods will yield insights into the transformation of metabolites as they transect the gut, and shed light on what bacterial players are likely responsible. The inclusion of functional data such as metagenomics, metatranscriptomics, and metaproteomics, varied metabolite detection methods including GC–MS, along with in vitro culturing experiments will lead to a more holistic and mechanistic understanding of the complexities of how marine herbivorous fish successfully deconstruct algal biomass and lead to new applied technologies to convert marine resources into value added bioproducts including biofuels.

## Supplementary Information

**Additional file 1.** Morphological data of dissected nenue.

**Additional file 2.** Individual wild nenue samples for this study.

**Additional file 3.** Supplementary Methods.

**Additional file 4.** Box and whisker plots of total reads per sample for DNA extraction blanks ("Blank"), PCR no template negative controls ("NTC"), mock communities ("Mock"), and samples ("Fish_Gut").

**Additional file 5.** Microbial alpha diversity across nenue gut subsections.

**Additional file 6.** Boxplots of (A) observed bacterial ASVs, (B) Shannon Eveness, and (C) Shannon Diversity across the 8 nenue gut subsections. Horizontal lines indicate the median, boxes span the IQR, whiskers extend to 1.5*IQR, and letters above bars denote significant pairwise differences between gut subsections determined by lsmeans with a tukey adjustment.

**Additional file 7.** Dispersion results for microbial communities and metabolomes.

**Additional file 8.** Pairwise PERMANOVA results for microbial communities.

**Additional file 9.** Pairwise PERMANOVA results for metabolomes.

**Additional file 10.** Visualization of Procrustes errors between microbial communities (16S rRNA) and metabolomic sample ordinations. Points indicate 16S samples and triangles indicate metabolomic samples. Points are colored according to gut subsection. Arrows indicate residual error after rotation between paired 16S metabolomic samples after optimal NMDS rotations. Arrows are colored according to gut subsection.

**Additional file 11.** All microbial ASVs that responded significantly to gut subsection, their FASTA sequences, associated taxonomies, relative abundances in each sample, and the gut module each ASV is enriched in.

**Additional file 12.** Abundant microbial ASVs that responded significantly to gut subsection, their associated taxonomies, enriched modules from Fig. 4, and mean relative abundances in each gut subsection.

**Additional file 13.** Abundant metabolite features that responded significantly to gut subsection, their associated ClassyFire chemical groups, enriched modules from Fig. 6, and mean relative abundances in each gut subsection.

## Data Availability

Sequencing reads from the demultiplexed samples analyzed in this study have been deposited in the NCBI Sequence Read Archive (SRA) under the BioProject accession PRJNA819194. All LC–MS/MS data are publicly available and deposited in the MassIVE data repository (http://massive.ucsd.edu) under the accession number MSV000086348. The Feature Based Molecular Networking analysis can be accessed at: https://gnps.ucsd.edu/ProteoSAFe/status.jsp?task=36b779130c3a41e6b26f0ca841003a9c Scripts used to analyze the 16S rRNA gene sequence data and metabolomic data in R have been deposited in GitHub at https://github.com/wesleysparagon/KRuMBS_FishGut_KonaJune2019.

## Abbreviations

ASV: Amplicon sequence variant
BSH: Bile salt hydrolase
GC-MS: Gas chromatography-mass spectrometry
GNPS: Global natural products social molecular networking
LC–MS/MS: Liquid chromatography-tandem mass spectrometry
NELHA: Natural energy laboratory of Hawaiʻi authority
NMDS: Non-metric multidimensional scaling
PCR: Polymerase chain reaction
PERMANOVA: Permutational analysis of variance
SCFAs: Short chain fatty acids
