# Supplementary figures and images for: Fine scale transitions of the microbiota and metabolome along the gastrointestinal tract of herbivorous fishes
Source: Anim Microbiome. 2022 May 23;4:33. doi: 10.1186/s42523-022-00182-z (PMC9128220; doi:10.1186/s42523-022-00182-z)

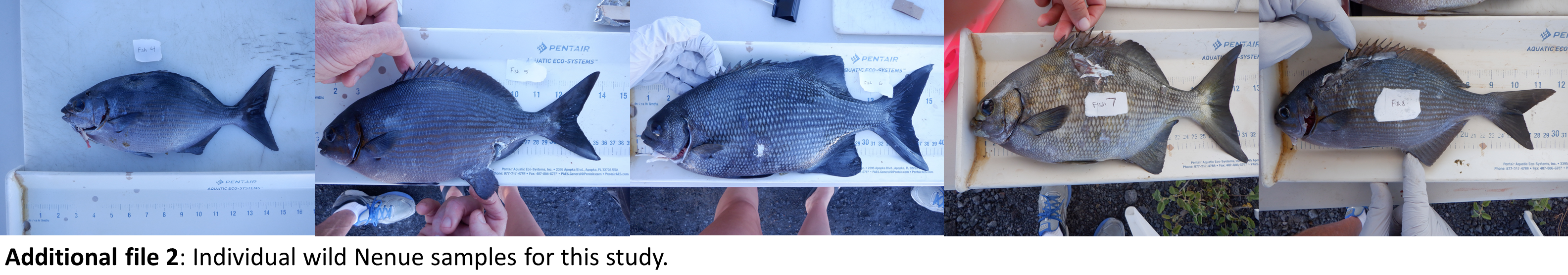

Supplement: Supplementary file 2 — Additional file 2. Individual wild nenue samples for this study. [file 42523_2022_182_MOESM2_ESM.png]

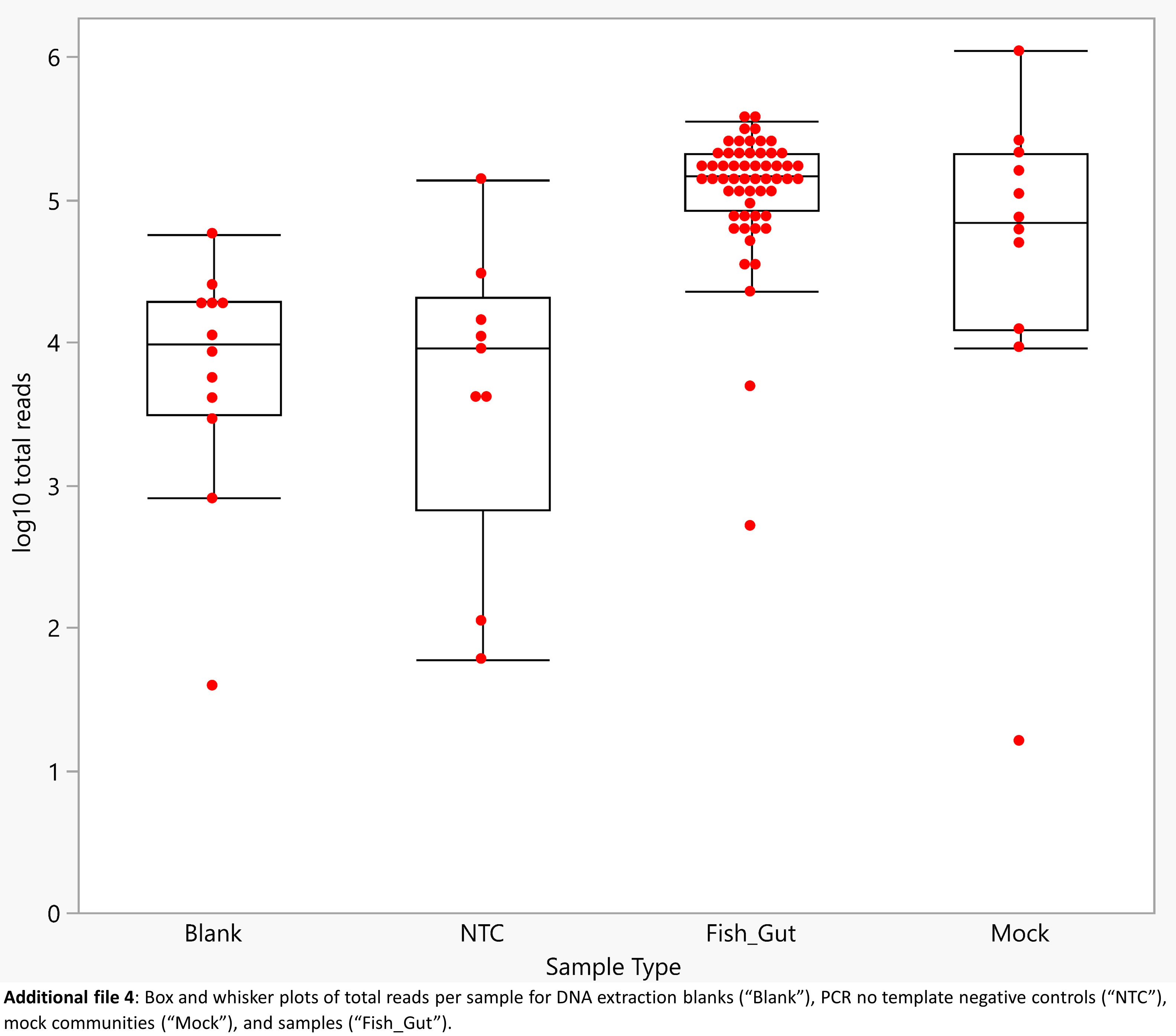

Supplement: Supplementary file 4 — Additional file 4. Box and whisker plots of total reads per sample for DNA extraction blanks ("Blank"), PCR no template negative controls ("NTC"), mock communities ("Mock"), and samples ("Fish_Gut"). [file 42523_2022_182_MOESM4_ESM.png]

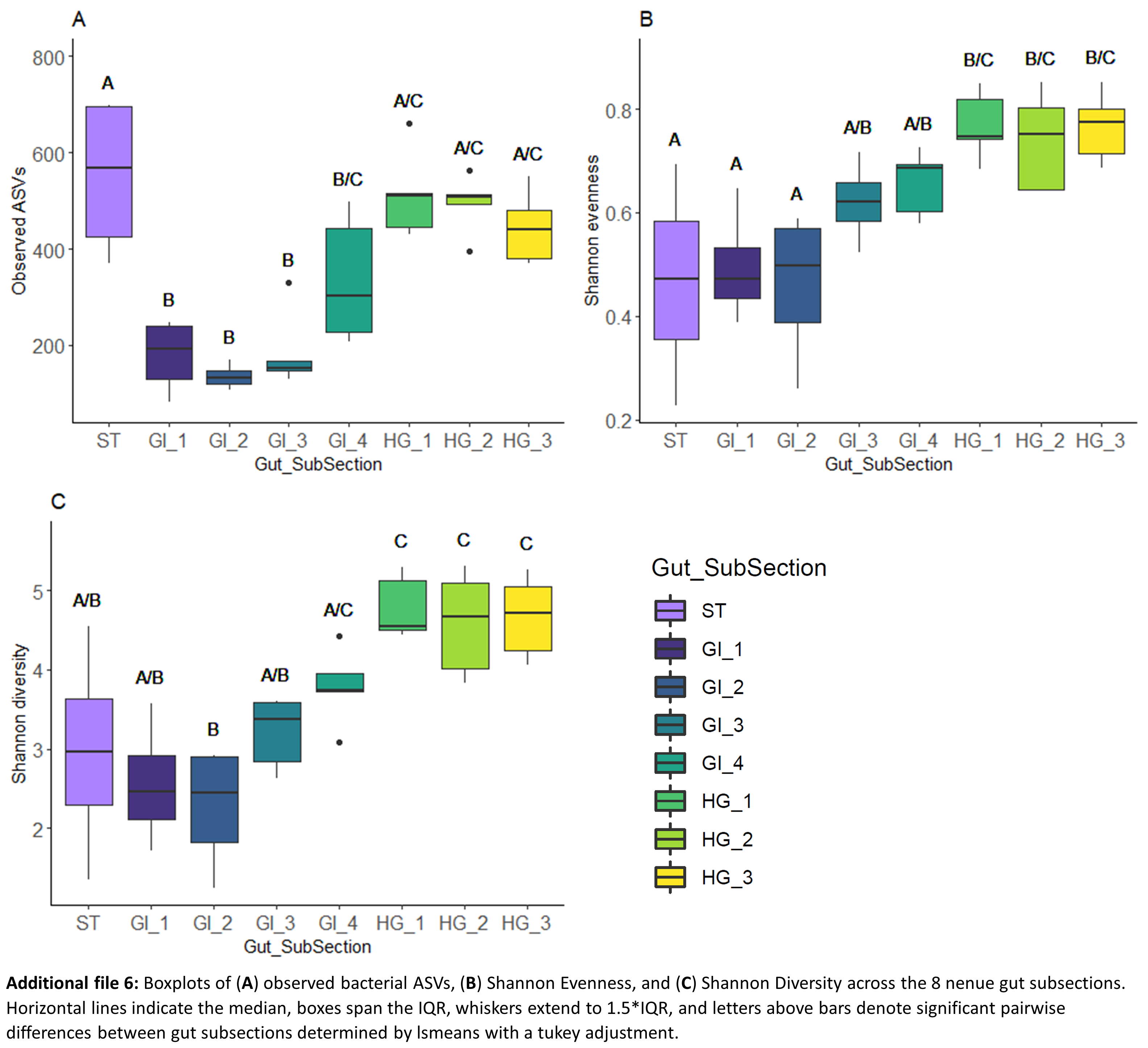

Supplement: Supplementary file 6 — Additional file 6. Boxplots of (A) observed bacterial ASVs, (B) Shannon Eveness, and (C) Shannon Diversity across the 8 nenue gut subsections. Horizontal lines indicate the median, boxes span the IQR, whiskers extend to 1.5*IQR, and letters above bars denote significant pairwise differences between gut subsections determined by lsmeans with a tukey adjustment. [file 42523_2022_182_MOESM6_ESM.png]

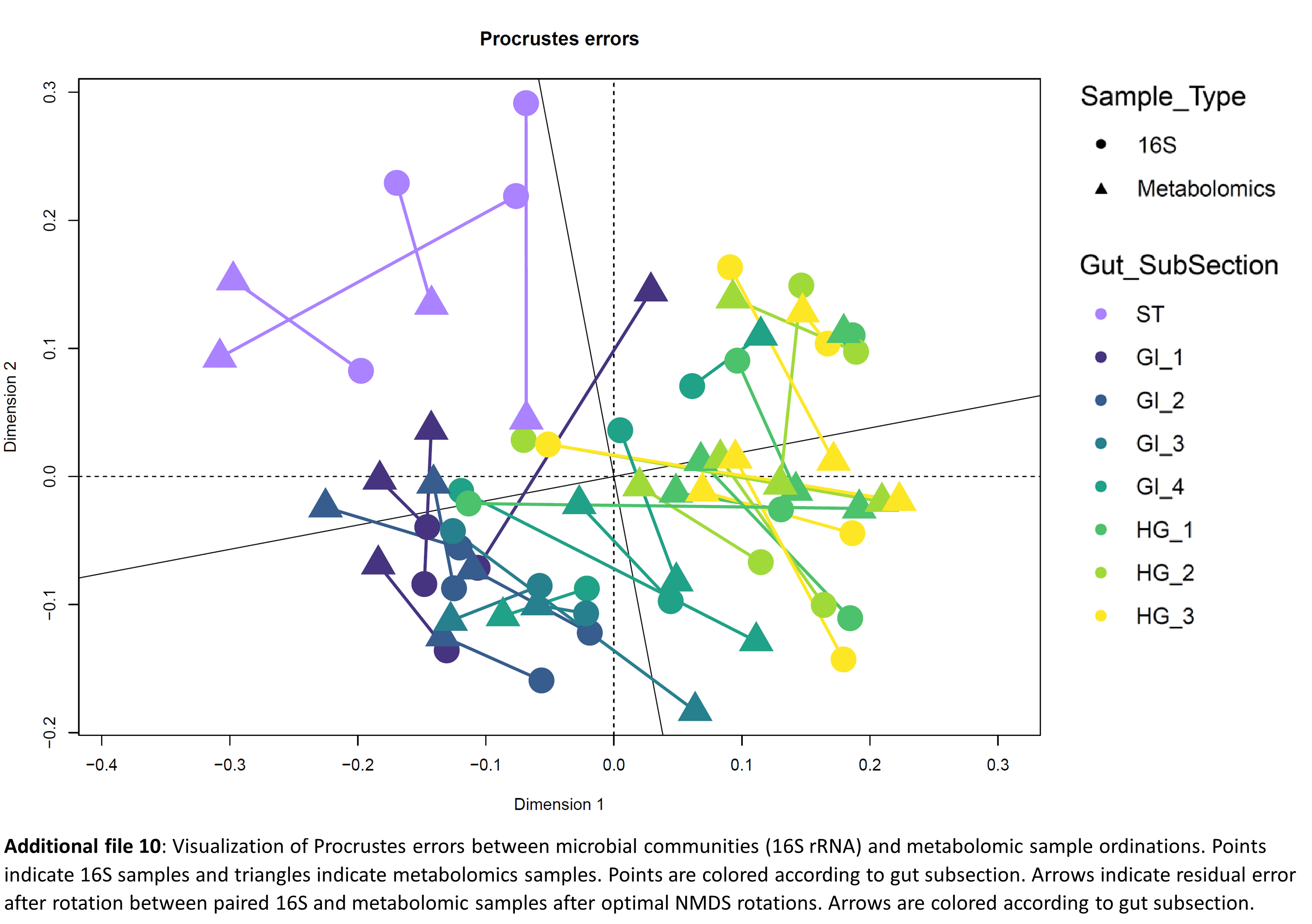

Supplement: Supplementary file 10 — Additional file 10. Visualization of Procrustes errors between microbial communities (16S rRNA) and metabolomic sample ordinations. Points indicate 16S samples and triangles indicate metabolomic samples. Points are colored according to gut subsection. Arrows indicate residual error after rotation between paired 16S metabolomic samples after optimal NMDS rotations. Arrows are colored according to gut subsection. [file 42523_2022_182_MOESM10_ESM.png]
