# Supplementary material for: Fine scale transitions of the microbiota and metabolome along the gastrointestinal tract of herbivorous fishes
Source: Anim Microbiome. 2022 May 23;4:33. doi: 10.1186/s42523-022-00182-z (PMC9128220; doi:10.1186/s42523-022-00182-z)
Supplement: Supplementary file 3 — Additional file 3. Supplementary Methods. [file 42523_2022_182_MOESM3_ESM.docx]

**Supplementary Methods**

PCR Conditions:

PCR reagents included 11.125 ul DNase free water, 0.25 ul 100X SYBR Green-I, 5 ul 5x buffer A (KAPA2G Robust Hot Start PCR Kit), 5 ul 5x Enhancer (KAPA2G Robust Hot Start PCR Kit), 0.5 ul dNTP 10 mM (KAPA2G Robust Hot Start PCR Kit), 1 ul Forward primer 10 uM, 1 ul Reverse Primer 10 uM, 0.125 ul taq (5U/ul KAPA2G Robust Hot Start PCR Kit Taq), and 1 ul DNA template. PCR was performed on an Eppendorf Mastercycler qPCR system using an initial denature of 95⁰C for 3:00 min followed by 35 cycles of 95°C for 30 seconds, 55°C for 45 seconds, 72°C for 90 seconds, followed by a final extension at 72°C for 5 minutes, a melt curve at 95°C over 12 minutes, and a final cycle of 95°C for 30 seconds, 55°C for 45 seconds, 72°C for 90 seconds, and extend at 72°C for 5 minutes.

16S Amplicon Sequencing Bioinformatics:

Raw paired fastq reads were preprocessed using the dada2 R package (Callahan et al. 2016). We truncated reads at position 220 (190 for the reverse read) and discarded them if they contained a number of expected errors above 3 using the filterAndTrim() function. Denoising was performed with the learnError() and dada() functions with default parameters. Using the mergePairs() function, we merged reads if they overlapped by at least 20 bases, and allowed for 1 mismatch at most. Triplicate technical replicates were then merged bioinformatically. We used mothur (Schloss 2009) along with the Silva (release 132) database (Quast et al. 2013) to align and annotate the sequences. Sequences with a start or stop position outside the 5th-95th percentile range (over all sequences) were discarded. We removed potential chimeras with chimera.vsearch() . Taxonomies were assigned using classify.seqs() and classify.otus(). We removed all mitochondrial or chloroplast OTUs, as well as sequences with no annotations at the domain level. Using sub.sample(), we normalized the abundance in each sample by subsampling 50,000 sequences from each. Samples with less than this amount were discarded. OTUs were defined as unique “amplicon sequence variants” by dada2; we used the lulu R package to refine OTUs: We merged two OTUs if all of the 3 following conditions were satisfied: 1) They co-occur in every sample, 2) One of the two OTUs has a lower abundance than the other if every sample and 3) they share a sequence similarity of at least 97% (Frøslev et al. 2017). Finally, we discarded OTUs with a total abundance of 2 or less.

Metabolomics Data Pre-processing with MZmine2:

Untargeted LC-MS/MS data pre-processing was performed with MZmine 2.37, corr17.7 version available at https://github.com/robinschmid/mzmine2/releases. (Katajamaa et al. 2006, Pluskal et al. 2010, Schmid et al. 2021). Intensity thresholds of 1E4 and 50 were for used for MS1 and MS2, respectively. Chromatograms were built using the ADAP chromatogram builder with a min group size of 3, group intensity threshold of 1E4, minimum peak intensity of 3E4, and m/z tolerance of 0.01 Da or 20 ppm. Extracted Ion Chromatograms (XICs) were deconvoluted using the local minimum search algorithm with a chromatographic threshold of 1%, a search minimum in RT range of 0.2 min, and a median m/z center calculation with m/z range for MS2 pairing of 0.01 and RT range for MS2 scan pairing of 0.2. Isotope peaks were grouped and features from different samples were aligned with 0.01 Da or 20 ppm mass tolerance and 0.3 min retention time tolerance. MS1 peak lists were joined using an m/z tolerance of 0.01 Da or 20 ppm and retention time tolerance of 0.3 min. Alignment was then performed by placing a weight of 75 on m/z and 25 on RT. The feature table of peak areas were exported as a .csv file and the corresponding consensus MS/MS spectra were exported as a .mgf file.

References:

Pluskal, T., Castillo, S., Villar-Briones, A. & Orešič, M. MZmine 2: Modular framework for processing, visualizing, and analyzing mass spectrometry-based molecular profile data. BMC Bioinformaticsvolume 11, 395 (2010).

Katajamaa, M., Miettinen, J. & Oresic, M. MZmine: toolbox for processing and visualization of mass spectrometry based molecular profile data. Bioinformatics 22, 634-636 (2006).

Schmid, R. et al. Ion identity molecular networking for mass spectrometry-based metabolomics in the GNPS environment. Nature Communications 12, 3832 (2021).
